# *Lacticaseibacillus rhamnosus*-Derived Exopolysaccharide Attenuates D-Galactose-Induced Oxidative Stress and Inflammatory Brain Injury and Modulates Gut Microbiota in a Mouse Model

**DOI:** 10.3390/microorganisms10102046

**Published:** 2022-10-17

**Authors:** Manorama Kumari, Vaishali L. Dasriya, Basavaprabhu H. Nataraj, Ravinder Nagpal, Pradip V. Behare

**Affiliations:** 1 Technofunctional Starter Lab., National Collection of Dairy Cultures (NCDC), Dairy Microbiology Division, National Dairy Research Institute, Karnal 132001, Haryana, India; 2 College of Dairy and Food Technology, Agriculture University, Jodhpur 342304, Rajasthan, India; 3 Department of Nutrition and Integrative Physiology, Florida State University, Tallahassee, FL 32306, USA

**Keywords:** exopolysaccharide, antioxidant, anti-inflammatory, gut microbiota, microbiota–gut–brain axis

## Abstract

This study aimed to investigate the protective effect of a novel exopolysaccharide EPSRam12, produced by *Lacticaseibacillus rhamnosus* Ram12, against D-galactose-induced brain injury and gut microbiota dysbiosis in mice. The findings demonstrate that EPSRam12 increases the level of antioxidant enzymes superoxide dismutase, catalase and glutathione peroxidase, total antioxidant capacity, and the level of anti-inflammatory cytokine IL-10, while decreasing malonaldehyde, nitric oxide, pro-inflammatory cytokines including TNF-α, IL-1β, IL-6, MCP-1, and the mRNA expression of cyclooxygenase-2, inducible nitric oxide synthase, and the activation of nuclear factor-kappa-B in the brain tissues of D-galactose-treated mice. Further analyses reveal that EPSRam12 improves gut mucosal barrier function and increases the levels of short-chain fatty acids (SCFAs) in the intestine while restoring gut microbial diversity by enriching the abundance of SCFA-producing microbial genera *Prevotella*, *Clostridium*, *Intestinimonas*, and *Acetatifactor* while decreasing potential pathobionts including Helicobacter. These findings of antioxidative and anti-inflammatory effects in the brain and ameliorative effects on epithelial integrity, SCFAs and microbiota in the gut, provide novel insights into the effect of EPSRam12 intervention on the gut–microbiome–brain axis and should facilitate prospective understanding of microbial exopolysaccharide for improved host health.

## 1. Introduction

Oxidative stress is induced by an unequal distribution in the formation of reactive oxygen species (ROS) and the potential of antioxidant systems to readily detoxify these reactive intermediates. Excessive and uncontrollable free radical production under oxidative stress conditions damages DNA, proteins, and lipids, compromising cell health and contributing to disease development [1,2,3]. The brain has lower antioxidant activity and is thought to be more vulnerable to oxidative damage than other body tissues [4].

D-galactose has been used as an oxidative stress inducer in animal models [5]. D-galactose oxidase is an enzyme that breaks down excessive amounts of D-galactose into aldose and hydrogen peroxide. The products then generate ROS via oxidative metabolism and glycosylation, resulting in oxidative stress, which activates NF-κB that is intimately linked to its transcriptional action on pro-inflammatory molecules, leading to inflammation [6]. Oxidative stress and inflammation are common factors responsible for neurological disorders, which, in turn, affect the gut microbial population [7]. Inflammation in the colon increases interferon gamma (IFN-γ) production, which causes phagocytic-innate immune cells to produce ROS. Reactive oxygen species such as superoxide, peroxide, and hypochlorite eventually form products, including DMSO (dimethyl sulfoxide), and TMAO (trimethylamine N oxide) for anaerobic respiration, which outgrow the growth of facultative anaerobes, resulting in a decrease in bacterial diversity [8]. As evidence mounts that changes in the bidirectional relationship between the gut and the brain are linked to the pathogenesis of gastrointestinal and neurological disorders, the microbiota–gut–brain axis has emerged as a new and widely accepted concept [9]. Therefore, an excessive dose of D-galactose has been used to establish animal models that not only cause oxidative and inflammatory stress in the brain but also cause gut microbiota dysbiosis [10].

Diet has a significant impact on microbiota diversity and composition, selectively influencing beneficial gut bacteria and host-microbe interactions [11]. For example, some dietary oligosaccharides or polysaccharides known as ‘prebiotics’, promote the growth and activity of specific beneficial microbes in the gut [12]. Furthermore, there has been a growing emphasis on the role of dietary habits in modulating the bidirectional interaction between gut microbiota and the brain by diets and prebiotics, which promote optimal brain health [13,14]. Short-chain fatty acids (SCFAs) are the main metabolites produced in the colon by the gut microbiota from dietary components (prebiotics) that are not absorbed or digested in the small intestine, and are speculated to play a key role in microbiota–gut–brain crosstalk [15]. The most prevalent SCFAs in the colon are acetic acid (C2), propionic acid (C3), and butyric acid (C4), which together account for 90–95% of the total amount [16]. Short-chain fatty acids are an important source of energy for intestinal epithelial cells, and are known to strengthen gut barrier function transmembrane proteins such as claudins, Occludin, and Zonula Occludens-1 (ZO-1) that are often thought to regulate intestinal permeability [17]. The integrity of the intestinal barrier is primarily associated with the controlled passage of molecules and nutrients from the circulation to the brain, which plays a critical role in brain health [15]. The SCFAs activate G protein-coupled receptors (GPR43 for acetate and propionate or GPR41 for other SCFAs), which are expressed in many sites, including the central nervous system, and play an important role in the regulation of metabolic homeostasis, inflammation, neurological disorders, and other diseases [14]. Therefore, dietary oligosaccharides or polysaccharides may prevent or delay brain oxidative and inflammatory stress as well as modulate the gut microbiota, influencing the microbiota–gut–brain axis.

Exopolysaccharide (EPS), a biopolymer, is becoming increasingly popular among the bioactive molecules produced by lactic acid bacteria (LAB). Exopolysaccharide is very attractive for the industrial sector due to its natural origins, sustainability, economic importance and health beneficial properties. The peculiar structural feature of EPS is likely to influence its biological activity and may modulate gut microbiota in the gastrointestinal tract. The electron-donating or electron-withdrawing functional groups and the reducing sugar monosaccharides of EPS have been determined to be the dominant property for certain antioxidant functions [18]. The stimulation of macrophage by EPS to reduce inflammation through inhibiting the NF-κB signaling pathway and reducing pro-inflammatory cytokines has been identified as an inflammatory property of EPS [19]. The fermentation of EPS by specific bacteria may result in the formation of short-chain fatty acids (SCFAs), which have a variety of beneficial effects in the host, including providing energy for colonocytes, maintaining the intestinal barrier, antioxidant, anti-inflammation, and modulating host metabolism and immune responses [20]. Although some LAB-derived EPSs have been reported for antioxidant and anti-inflammatory properties, the effect of such EPSs on oxidative and inflammatory stress in the brain and on epithelial and microbiome arrays in the gut in the same settings have rarely been investigated. Accordingly, detailed in vivo studies to evaluate the anti-oxidative and anti-inflammatory effects in brain and gut modulation potency of EPS as well as its influence on the microbiota–gut–brain axis are indispensable. To this end, we herein develop and evaluate EPSRam12 produced from *Lacticaseibacillus rhamnosus* Ram12 (NCDC710), a high molecular weight, branched-chain heteropolysaccharide with α-configuration, with considerable in vitro antioxidant and immunomodulatory properties (unpublished data), as a novel biomolecule that could influence the gut–microbiota–brain axis by ameliorating the oxidation and inflammation in the brain, strengthening intestinal epithelial barrier integrity, as well as modulating microbiome composition and SCFA production in the gut.

## 2. Materials and Methods

### 2.1. Production and Extraction of Exopolysaccharides 

Exopolysaccharide-producing *Lacticaseibacillus rhamnosus* (previously, *Lactobacillus rhamnosus*) strain Ram12 (NCDC710) was procured from the National Collection of Dairy Cultures (NCDC), ICAR-National Dairy Research Institute, Karnal, Haryana, India. The strain was cultured and propagated in sterile De Man, Rogosa, and Sharpe (MRS) broth at 37 °C for 18–24 h. To produce EPS, *L. rhamnosus* Ram12 was cultured in the previously optimized deproteinized whey medium [21]. The culture supernatant was collected and precipitated by repetitive ethanol extraction [22]. The precipitated crude EPS obtained after centrifugation at 8000× *g* for 20 min was lyophilized and was named as EPSRam12. The yield of EPSRam12 (g/L) on wet basis, (mg/L) on dry basis, and (mg/mg of bacterial cell) on dry basis was determined.

### 2.2. Animal Model and Treatment

All animal experiments were performed in accordance with the Committee for the Purpose of Control and Supervision of Experiments on Animals’ (CPCSEA) Guidelines on the Regulation of Scientific Experiments on Animals. The study was approved by Institutional Animal Ethics Committee (IAEC), National Dairy Research Institute, India (NDRI-IAEC Approval No. 46-IAEC-20-37 dated 22 July 2020). Special efforts were made to minimize discomfort to animals. Forty-eight male Swiss albino mice were procured from Small Animal House of National Dairy Research Institute (NDRI), Karnal, Haryana, India. The animals were housed in polycarbonate cages, with food and water provided ad libitum under a 12-h light/dark cycle at a temperature of 24 ± 2 °C and humidity 55–60%. After 1 week of acclimatization, 48 male mice (12-week-old, 25–28 g of body weight) were randomly divided into six groups (eight mice per group): Control group, no treatment; D-galactose group, daily treated with an intraperitoneal injection of D-galactose of 100 mg/kg body weight (BW); Stress-control group, daily treated with an intraperitoneal injection and oral gavage of 0.1 M Phosphate Buffer Saline (PBS, pH 7.2); Beta-glucan group (positive control group), daily treated with an intraperitoneal injection of D-galactose of 100 mg/kg BW and with oral administration of 25 mg/kg BW of Betaglucan; EPS25 group (low dose EPS), daily treated with an intraperitoneal injection of D-galactose of 100 mg/kg BW and with oral administration of 25 mg/kg BW of EPSRam12; and EPS50 group (high-dose EPS), daily treated with an intraperitoneal injection of D-galactose of 100 mg/kg BW and with oral administration of 50 mg/kg BW of EPSRam12 for 45 days. Mice were sacrificed at the end of treatment (after 45 days), and tissues were immediately collected for further experiments. Each hippocampus tissue was collected and homogenized with ice-cold 50 mM PBS (pH 7.4) and centrifuged at 8000× *g* for 10 min at 4 °C to collect the supernatant. The supernatant was stored at −20 °C for further biochemical assay.

### 2.3. Histological Analysis

The brain and colon tissue were collected and fixed in 10% formalin solution for 12 h. The samples were dehydrated by immersion in xylene, embedded in paraffin, cut into 4-μm thick section, stained with haematoxylin and eosin (HE), and finally examined under light microscopy.

### 2.4. Determination of SOD, CAT, GSH-Px, TAC, MDA and NO

The activities of superoxide dismutase (SOD), catalase (CAT), glutathione peroxidase (GSH-Px), and total antioxidant capacity (TAC) in hippocampus homogenates was measured through the available commercial kits (BT Bioassay, Shanghai, China) according to the manufacturer’s protocol. The absorbance was measured in 450 nm wavelength using a microplate reader (BioTeK, Winooski, VT, USA). Malonaldehyde (MDA) was estimated as per the protocol given by [23]. Briefly, 10% in hippocampus homogenates was mixed with sodium dodecyl sulphate, acetate buffer (pH 3.5), and thiobarbituric acid aqueous solution. The red pigment produced after heating at 95 °C for 60 min was extracted with an n-butanol–pyridine mixture and estimated using the absorbance at 532 nm. Tetramethoxy-propane (malonaldehyde bis(dimethyl acetal)) (Sigma, St. Louis, MO, USA) was used as an external standard, and the lipid peroxide level was expressed in nmol malondialdehyde. The measurement of nitric oxide (NO) production in hippocampus homogenates was measured by indirect spectrophotometric method using Griess reaction [24]. Briefly, a microplate reader (BioTeK, USA) was used to measure the absorbance at 550 nm after 45 min of incubating equal volumes (100 μL) of supernatant and Griess reagent (Sigma, USA) at room temperature. As a standard, sodium nitrite was employed and the level was expressed in µmol nitric oxide.

### 2.5. Measurement of IL- IL-6, IL-1β, TNF-α, MCP-1, and 1L-10

Quantitative detection of interleukin-6 (IL-6), interleukin-1β (IL-1β), tumor necrosis factor-alpha (TNF-α), interleukin-10 (IL-10), and monocyte chemoattractant protein-1 (MCP-1) in hippocampus homogenates was performed by using commercially available ELISA kit (BT Bioassay, China), according to the manufacturer’s protocol. The absorbance was measured in 450 nm wavelength using a microplate reader (BioTeK, USA).

### 2.6. Measurement of Lipofuscin Content

Lipofuscin (LF) content in the brain was evaluated following the previously described methods [25]. Briefly, an extraction mixture made of chloroform and methanol (2:1, *v*/*v*) was combined with 2 mL of brain homogenate. For 10 min, the solution was centrifuged at 3000 rpm. The top layer was aspirated, and the final volume was then adjusted to 5 mL by adding a chloroform–methanol mixture. A UV-VIS Spectrophotometer (UV 1800, Shimadzu, Kyoto, Japan) was used to measure the absorbance at maximum excitation and maximum emission wavelengths of 365 nm and 435 nm, respectively. A fresh solution of quinine bisulphate (1 µg/mL) was standardized for fluorescence intensity. The wet brain’s LF content was used to express the experimentally determined findings. 

### 2.7. Effects of EPSRam12 on Intestinal Tight-Junction and Inflammatory Gene Expression by RT-qPCR

Effects of EPS on the mRNA expression levels of intestinal tight-junction genes viz. zonulae occludens (ZO-1), Occludin, and Claudin-1 as well as on p65, COX-2, and iNOS gene expression in the hippocampus of the brain were analyzed by reverse transcription quantitative real-time PCR (RT-qPCR) following the method described previously [26]. Briefly, total RNA samples were extracted from the distal colon to analyze the expression of ZO-1, Occludin, and Claudin-1, and from the hippocampus of the brain to analyze the expression of p65, COX-2, and iNOS, with Aurum™ Total RNA Mini Kit (Bio-Rad, Hercules, CA, United States) according to the manufacturer’s instructions. The RNA concentration and quality were evaluated with spectrophotometer SYNERGY H1 Hybrid Reader (BioTEK, USA) and 1.2% agarose-gel electrophoresis, respectively. The total RNA from each sample was reverse transcribed to cDNA with the Thermo Scientific Revert Aid First Strand cDNA Synthesis Kit (Thermo Scientific, Waltham, MA, USA), according to the instructions of the manufacturer. Real-time quantitative PCR was performed with SYBR™ Green PCR Master Mix (Thermo Scientific) in a CFX96 Touch Real-Time PCR Detection System (Bio-Rad). The primers for real-time amplification were analyzed as per the primer sequence given in Table 1, [26]. The PCR cycling programmed was: 95 °C for 3:00 min; 39 cycles of 95 °C for 10 s and 58 °C for 30 s. Data were examined using the comparative threshold cycle (Cq) method and normalized to β-actin and GAPDH, two endogenous references or housekeeping genes. The 2^−ΔΔCT^ method was used to calculate the target transcript’s quantities in relation to the selected reference gene transcript.

### 2.8. Microbiome Analysis

Genomic DNA was extracted from cecal samples of each mouse in all six groups (control, Stress-control, Beta-glucan, EPS25, EPS50, D-galactose) using Quick-DNA Cecal/Soil Microbe DNA Miniprep Kit (Zymo Research, Irvine, CA, USA) after optimizing the bead-beating inFastPrep-24 bead homogenizer (MP Biomedicals LLC, Solon, OH, USA)) conditions. The concentration and purity of metagenomic DNA samples was determined using SYNERGY H1 Hybrid Reader (BioTEK, USA). The high purity cecal metagenomic DNA extracted from all the mice in each group was pooled for each group. 16S MicroBiome Profiling of the 16S rRNA V3-V4 hypervariable region of cecal samples was performed using Illumina MiSeq platform to compare the microbiota diversity and composition across the cecal DNA samples of six groups (control, Stress-control, Beta-glucan, EPS25, EPS50, D-galactose). The obtained sequences (.fastq files) were processed with the QIIME2 (version 2.2021.2; https://qiime2.org/ (accessed on 12 October 2021)) bioinformatics pipeline [27], in a miniconda environment as per our previously described methods [28,29,30,31], followed by quality-filtering, adapter-trimming, denoising, and removal of non-chimeric amplicons by using the dada2 pipeline [32,33] using default parameters, as per our previous reports [29,30]. To avoid the bias if any of the sequencing depth, alpha-rarefaction was executed at the lowest sequencing depth. Amplicon sequence variants (ASVs) were assigned bacterial taxonomic nomenclature by using the Naive Bayes classifier natively installed in dada2 workflow and pre-trained with SILVA reference database (version 138.1; https://www.arb-silva.de/ (accessed on 12 October 2021)) with 99% sequence identity threshold [33], as described earlier [29,30]. The obtained sequence reads were filtered to exclude features annotated as ‘mitochondria’ and ‘chloroplast’. Community dissimilarity (β-diversity) was estimated by weighted and unweighted unifrac distance within qiime2 and visualized by PCoA (principal coordinate analysis). Alpha-diversity was calculated in the form of Chao1 and Shannon indices. The raw read counts obtained were finally transformed to the corresponding relative abundances by dividing each value by the total reads per sample followed by collapsing to taxonomic levels by summing their corresponding relative abundance. Obtained data were analyzed using the ‘R’ statistical software package (version 4.1.2; R Core Team, https://www.R-project.org/ (accessed on 12 October 2021)).

### 2.9. Cecal SCFA Levels 

The SCFAs from each mouse from the six groups (control, stress-control, Beta-glucan, EPS25, EPS50, D-galactose) were extracted by previously described method [34] with slight modification. Briefly, 300 mg of mice cecal sample was vortex with 500 µL of water and mixed with 1 mL of 25% meta-phosphoric acid. The mixed samples were incubated overnight at refrigeration temperature. After the incubation, the samples were centrifuged at 10,000 rpm for 10 min at 4 °C, and the supernatant was collected. The collected supernatant was analyzed for SCFAs using Gas Chromatography (GC) (5765 Nucon, New Delhi, India) and a N_2_ flame ionization detector was used. The separation of SCFAs was achieved with Chromosorb 101 column under operation conditions as follows: injection volume was 1 μL, injection port temperature was 200 °C, column temperature 145 °C, carrier gas was N_2_, detector temperature was 180 °C. Each GC run for the determination of samples lasted 10 min. The standard curve of acetate, propionate, and butyrate was prepared for the quantification of SCFAs in cecal samples of mice. 

### 2.10. Statistical Analysis

The results of each experiment were carried out in triplicate and are presented as mean ± SEM (standard error mean). Analysis of Variance (ANOVA), one-way or two-way wherever appropriate, followed by Bonferroni post-test, was performed using GraphPad Prism (version 5). Microbiome analyses including diversity, composition, and heatmap clustering were performed using ‘R’ statistical package (ver. 4.1.2) using ‘vegan’ and ‘ggplot’ libraries. *p* < 0.05 was considered statistically significant.

## 3. Results and Discussion

### 3.1. Yield of EPSRam12

The yield of EPSRam12 was found to be 2.1 g/L on wet basis, 253 ± 13.5 mg/L on dry basis, and 0.76 ± 0.21 mg/mg of bacterial cell on dry basis. A previous study [35] reported 1.038 g of EPS in culture supernatant per gram of bacterial cells (dry weights) produced by *Limosilactobacillus reuteri* strain 100-23. This variation in the yield of EPS production could be ascribed to the effect of processing conditions, growth media, type of culture, and method of EPS extraction as previously reported [21]. 

### 3.2. Effects of EPSRam12 on Histopathology

**Figure 1:**
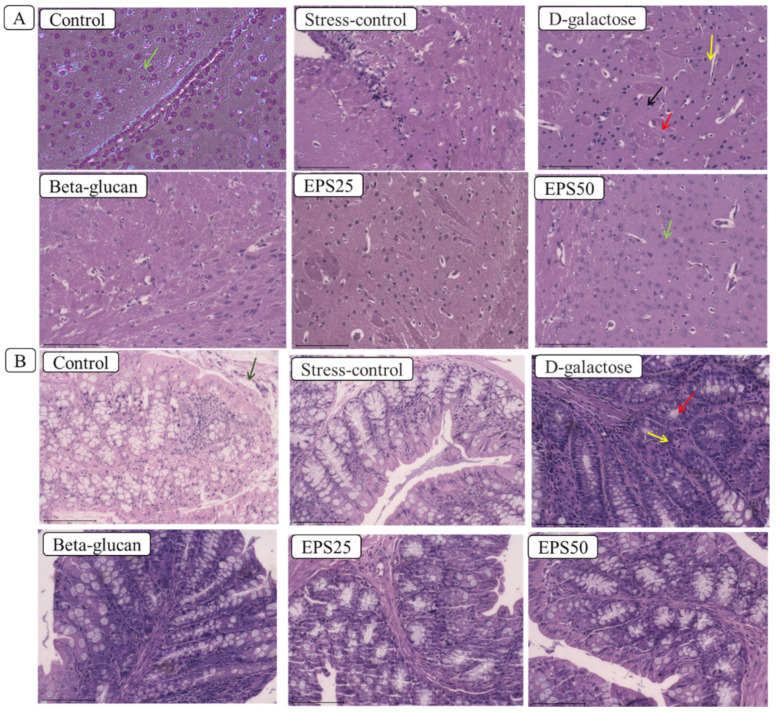
Effects of EPSRam12 on morphology of brain and colon tissue in D-galactose-induced mice. (**A**) Images of mouse brain slices stained with H and E: (Yellow arrow—disorganized nerve fibers, black arrow—irregular and reduced neuron number, red arrow—vacuolar changes in cytoplasm); (**B**) images of mouse colon slices stained with H and E: (Green arrow—intact epithelial layer architecture, red arrow—goblet cell depletion, yellow arrow—inflammation with infiltrating leukocytes).

The histopathological examination of brain and colon tissues was used to determine the protective effect of EPSRam12 on D-galactose-induced injury (Figure 1). The histopathological examination of the brain showed the absence of any pathological changes in control group, while a typical brain tissue injury characteristic such as disorganized nerve fibers (yellow arrow) with irregular and reduced neuron number (black arrow), and vacuolar changes in cytoplasm (red arrow) was observed in D-galactose group (Figure 1A). 

However, after EPSRam12 intervention, there was a noticeable increase in round-shaped neurons and well-organized fibers (green arrow). The morphological structure of the EPS (50 mg/kg) group appeared very similar to that of the control and positive control groups (Figure 1A), indicating that EPS treatment could improve the extent of pathological changes in brain tissues to varying degrees. In line with this finding, a previous study [36] reported reduced cell number and disordered arrangement of hippocampal neurons in the D-galactose model group. The finding of fewer cerebral cortical nerve cells, decreased number of pyramidal cells, sparse and irregular arrangement, and decreased cell volume and nuclear pyknosis was also reported in the D-galactose model group [37]. 

The colon of control mice had normal and intact epithelial layer architecture (green arrow) and no loss of crypt cells (Figure 1B), whereas, the D-galactose group had relatively damaged epithelial architecture, goblet cell depletion (red arrow), inflammation with infiltrating leukocytes (yellow arrow), and a very thin muscular layer, compared to the control group (Figure 1B). The abnormal epithelial and muscular layer structure, as well as goblet cell number, were improved in the EPSRam12 and Beta-glucan groups, while the high dose of EPS (EPS50) could better ameliorate the intestine damage (Figure 1B). Similarly, a previous study [25] reported sparse intestinal villi and structural damage, as well as a very thin muscular layer in the D-galactose group compared to the control group. It is clear from the above histopathology results that the injury caused by D-galactose can be alleviated with the supplementation of EPSRam12 and Beta-glucan, and a high dose of EPSRma12 (EPS50) acts superiorly, implying that EPSRam12 can significantly ameliorate injuries caused by D-galactose injection in a dose-dependent manner, and exert a protective effect on the brain and colon in D-galactose-induced-stress mice.

### 3.3. Effects of EPSRam12 on Oxidative Stress Markers 

**Figure 2:**
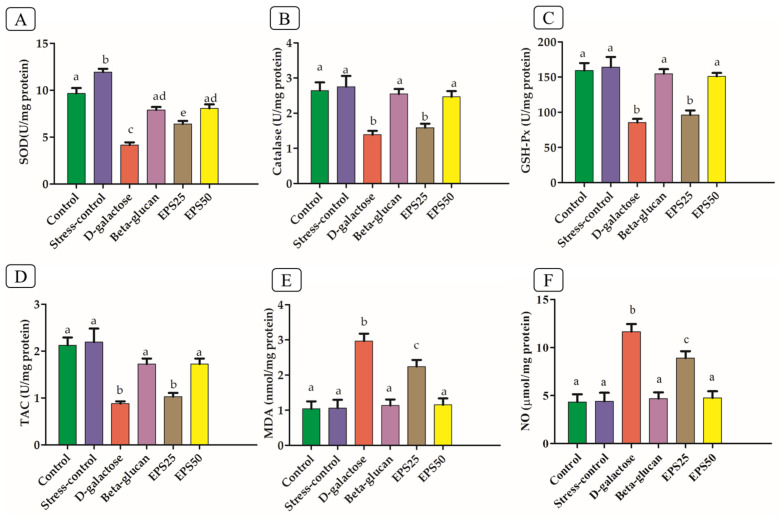
Effects of EPSRam12 on oxidative stress in the brain of D-galactose-induced mice. (**A**) Superoxide dismutase (SOD), (**B**) Catalase (CAT), (**C**) Glutathione peroxidase (GSH-Px), (**D**) Total antioxidant capacity (TAC), (**E**) Malonaldehyde (MDA), and (**F**) Nitric oxide (NO). Data are presented as mean ± SEM. Data were analyzed using One-way ANOVA with Bonferroni post-test. Values with dissimilar superscript alphabets (a–c) indicate significant differences (*p* < 0.05) among the treatment groups.

An excessive dose of D-galactose is known to induce oxidative stress, which produces ROS. The brain has lower antioxidant activity and is thought to be more vulnerable to oxidative damage than other body tissues [4]; therefore, antioxidants should be supplemented. Based on these assumptions, we evaluated the effect of EPSRam12 in the brain of the D-galactose-induced oxidative stress mouse model (Figure 2).

The D-galactose group showed a significant decrease (*p* < 0.05) in SOD, CAT, GSH-Px, and TAC activities and increase in MDA and NO level, compared with that in the control group, indicating the occurrence of oxidative stress in the brain. Similarly, a previous study [38] reported decreased CAT, GSH-Px, SOD, and TAC activities and increased levels of MDA and NO in the brain of D-galactose–induced mice. A significant increase (*p* < 0.05) in the SOD level (Figure 2) of the stress-control group as compared to the control is indicative of an increase in SOD to combat the ROS produced with stress caused by the handling of mice during treatment [39]. A significant increase (*p* < 0.05) in SOD but insignificant increase in CAT, GSH-Px, and TAC in the stress-control group after oral and intraperitoneal injection of isotonic saline solution (PBS solution) showed a mild stress-producing effect but not sufficient to induce a significant inflammatory response. The supplementation of EPSRam12 significantly improved SOD, CAT, GSH-Px, and TAC activities and decreased MDA and NO level (Figure 2). The high dose EPS (EPS50) group followed the same trend as the Beta-glucan (positive control) group, indicating that EPS supplementation could improve the antioxidant capacity of the brain in a dose-dependent manner. The SOD, CAT, and GSH-Px activities and TAC content increased to 46.61%, 14.38%, 12.49%, and 17.32%, when treated with EPSRam12 at the dose of 25 mg/kg bw, while the levels increased to 84.94%, 77.54%, 76.86%, and 96.45% at the dose of 50 mg/kg, respectively, when compared to that in the D-galactose group (Figure 2). The MDA and NO contents in the brains of D-galactose-induced mice were 3.52 ± 0.27 nmol/mg protein and 11.66 ± 0.79 µmol/mg protein, which were decreased to 2.47 ± 0.13 nmol/mg protein and 8.90 ± 0.71 µmol/mg protein when treated with EPSRam12 at the dose of 25 mg/kg, while the corresponding parameter showed lower level 1.15 ± 0.18 nmol/mg protein and 4.75 ± 0.68 µmol/mg protein when treated with EPSRam12 at the dose of 50 mg/kg, respectively (Figure 2). The insignificant effect of low-dose EPSRam12 in the prevention of oxidative stress in the brain of D-galactose-induced stressed mice suggests that EPSRam12 has a dose-dependent antioxidant activity. 

### 3.4. Effects of EPSRam12 on Inflammatory Markers 

**Figure 3:**
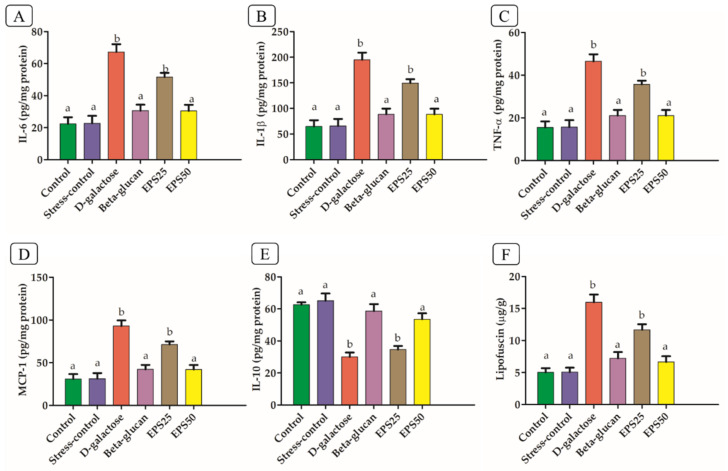
Effects of EPSRam12 on inflammatory markers and Lipofuscin content in the brain in D-galactose-induced mice. (**A**) IL-6, (**B**) IL-1β, (**C**) TNF-α, (**D**) MCP-1, (**E**) IL-10, and (**F**) Lipofuscin. Data are presented as mean ± SEM. Data were analyzed using One-way ANOVA with Bonferroni post-test. Values with dissimilar superscript alphabets (a–b) indicate significant differences (*p* < 0.05) among the treatment groups.

The upregulation of pro-inflammatory cytokines (IL-6, IL-1β, TNF-α, and MCP-1) and downregulation of anti-inflammatory cytokines (IL-10) in the brains of the D-galactose group was observed, compared with the control group (Figure 3), indicating the occurrence of inflammation. 

The increased ROS and their reaction products after the injection of D-galactose activate NF-κB, which is intimately linked to its transcriptional action on pro-inflammatory molecules such as TNF-α, IL-6 and IL-1β [40,41]. Several studies have reported an elevated level of pro-inflammatory cytokines and decreased level of anti-inflammatory cytokines with the injection of D-galactose [38,42]. The treatment with EPSRam12 and Beta-glucan significantly decreased pro-inflammatory cytokines (IL-6, IL-1β, TNF-α, and MCP-1) and increased anti-inflammatory cytokines (IL-10) compared with the D-galactose group (*p* < 0.05), indicating that EPSRam12 has a suppressive effect on the expression of hippocampal inflammatory cytokines. 

### 3.5. Effects of EPSRam12 on Lipofuscin Content

Lipofuscin (LF), regarded as aggregates of undigested cell materials, increased significantly in the brain (*p* < 0.05) of the D-galactose group, compared to the control. Long-term D-galactose administration has been reported to increase LF levels in mice compared to control mice [43]. Increased lipofuscin accumulation can cause protein damage, DNA replication, and RNA synthesis interruption and may promote the generation of ROS, sensitizing cells to oxidative injury via lysosomal destabilization, making cells significantly more vulnerable to oxidative stress [43]. Additionally, LF may activate macrophages to stimulate the release of pro-inflammatory chemokines and cytokines, leading to a chronic oxidative-inflammatory process [44]. However, the treatment with EPSRam12 and Beta-glucan significantly (*p* < 0.05) decreased the LF content compared with the D-galactose group. The content of LF was notably reduced in the high-dose EPSRam12 (EPS50) group (Figure 3F). The supplementation of self-made aged garlic extract has been reported to promote increased level of LF content [43]. 

### 3.6. Effects of EPSRam12 on Intestinal Tight-Junction and Brain Inflammatory Genes

**Figure 4:**
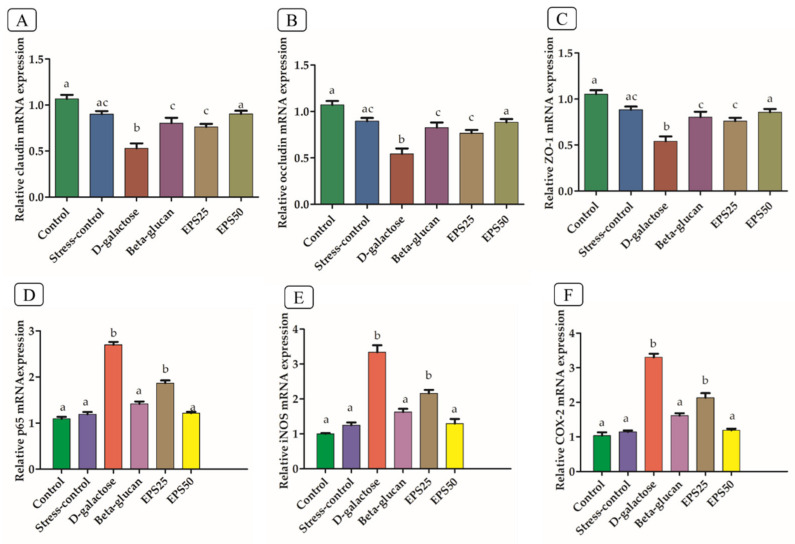
Effects of EPSRam12 on real-time quantitative PCR expression of tight-junction mRNA in colon and pro-inflammatory mRNA in brain of D-galactose-induced mice. (**A**) Claudin-1 mRNA, (**B**) Occludin mRNA, (**C**) ZO-1 mRNA, (**D**) p65 mRNA, (**E**) iNOS mRNA, and (**F**) COX-2 mRNA. Values are means ± SEMs. Data were presented as mean ± SEM. Data were analyzed using One-way ANOVA with Bonferroni post-test. Values with dissimilar superscript alphabets (a–c) indicate significant differences (*p* < 0.05) among the treatment groups.

As shown in Figure 4A–C, the expression levels of tight-junction (TJ) genes ZO-1, Claudin-1, and Occludin were observed to be significantly reduced (*p* < 0.05) in the colonic tissues of the D-galactose group, compared to the control group. The oxidative stress induced by the injection of D-galactose, which has been reported to disrupt the intestinal barrier function, decreased the expression of Claudin-1, Occludin, and E-cadherin in the D-galactose group, compared with the control group [45,46]. As a result, the regulation of epithelial cell barrier function via regulation of TJ gene expression and localization is a potential new target for the treatment of disease [47]. The supplementation of EPSRam12 counteracted the reduced expressions of ZO-1, Claudin-1, and Occludin (Figure 4A–C), in a dose-dependent manner, which indicates the protective effect of EPSRam12. Several studies have emphasized the importance of using specific EPS to strengthen barrier integrity. Heteropolysaccharide EPS, derived from *Streptococcus thermophilus* MN-BM-A01, has been reported to counteract the decreased colonic epithelial tight-junction expression of Claudin-1, Occludin, and E-cadherin caused in the Dextran sulfate sodium (DSS) mouse model [48]. EPS116, an exopolysaccharide from *Lactiplantibacillus plantarum* NCU116, has been reported to promote epithelial barrier function and TJ protein expression (Claudin-1, Occludin, and ZO-1) in DSS-induced colitis mice [49]. 

When compared to the control group, the brain tissues of the D-galactose group showed significantly higher expression of nuclear factor-kappa B (NF-κB p65) and thereby also enhanced expression of molecules that cause inflammation, such as cyclooxygenase-2 (COX-2) and inducible nitric oxide synthase (iNOS), which are primarily regulated by transcriptional factor NF-κB (Figure 4D–F). In line with this finding, a previous study [50] observed increased mRNA expression of COX-2 and iNOS as well as the activation of NF-κB in the brains of the D-galactose mice group, as compared to the control group. D-galactose is effective in increasing inflammatory markers and promoting neuroinflammation by activating the transcription factor NF-κB via redox-sensitive signaling pathways, resulting in memory damage [51]. As a result, therapeutic agents such as EPS that inhibit the expressions of pro-inflammatory molecules (COX-2, and iNOS), as well as reduce the activation of NF-κB, may be useful in reducing the severity of the inflammatory conditions. It is clear from Figure 4D–F that EPSRam12 significantly inhibited the upregulation of NF-κB p65, COX-2, and iNOS expression in the brain caused by the injection of D-galactose. This suggests that EPSRam12 may have therapeutic potential in the gut–brain axis by improving intestinal permeability and alleviating neuroinflammation.

### 3.7. Gut Microbiome Modulation in Mice Treated with EPSRam12

D-galactose treatment not only impairs brain function but also causes dysbiosis of the gut microbiota. Using a high-throughput 16S rRNA gene sequencing approach, we observed the protective effects of EPSRam12 on the gut microbiota dysbiosis caused by D-galactose. The effect of EPSRam12 on cecal microbiota α-diversity was evaluated by the number of ASVs and the Chao1 and Shannon indices. As shown in Table 2, there were no remarkable differences in the number of ASVs in the D-galactose group and control group, but increased ASVs in the EPS50 group and decreased ASVs in the Beta-glucan and EPS25 groups were observed. However, a higher Chao1 index was observed in the Beta-glucan and the D-galactose group, while a lower Chao1 index was observed in the EPS25, EPS50, and stress-control groups as compared to the control. In line with this result, previous studies have also observed a significant increase in the Chao1 index in the D-galactose-induced aged mice [38]. Whereas not entirely consistent with our findings, some reports have a found lower Chao1 index in naturally aging mice and D-galactose-induced aging mice [52,53]. This inconsistency of our result from previous studies may be explained by the fact that the Chao1 richness estimator is a non-parametric estimator that computes the lowest number of species in a sample, or in other words represents the richness of samples based upon the number of rare classes (i.e., ASVs or OTUs) that are found in a sample. When sample sizes are small, it underestimates true richness [54]. 

In contrast, Shannon’s diversity index has been reported to be appropriate for comparing environments when the number of sequences is normalized, even with low coverage [54]. The Shannon index represents both abundance (richness) and evenness, and an increase in its value indicates that samples have a greater species diversity. We observed a reduced Shannon index in the D-galactose group as compared with the control group (Table 2). A decrease in diversity caused by the injection of D-galactose is consistent with previous reports [52,53]. Moreover, it is clear from the result of the Shannon index that EPSRam12 supplementation effectively increased the Shannon index in a dose-dependent manner, compared with the D-galactose group, indicating that EPSRam12 supplementation could improve gut microbiota diversity and ameliorate dysbiosis in D-galactose-induced mice.

**Figure 5:**
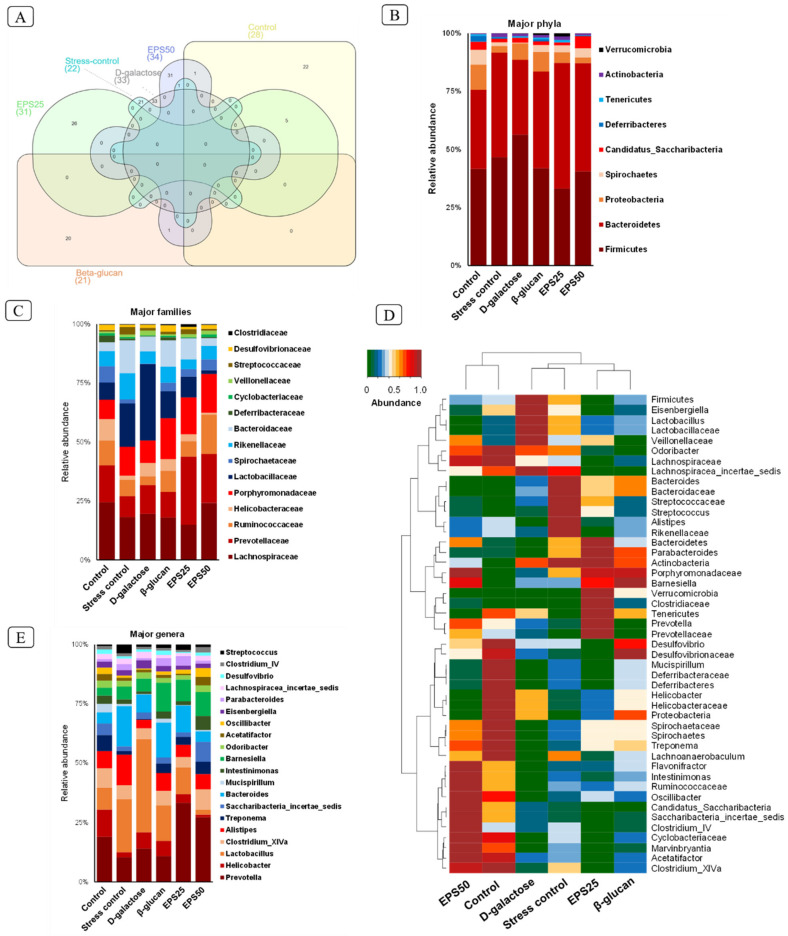
Distinct arrays of gut microbiota composition in different experimental groups of mice. (**A**) Venn diagram showing unique ASVs; (**B**,**C**,**E**) Microbiota composition at the level of major phyla, families and genera; and (**D**) hierarchical heat-map clustering analysis of major microbial taxa in different groups of mice.

The Venn diagram analysis of microbiome taxa showed 28, 22, 33, 21, 31, and 34 unique ASVs (Figure 5A) among 675, 579, 725, 797, 681, and 685 total ASVs in the control, Stress-control, D-galactose, Beta-glucan, EPS25, and EPS50 groups, respectively, suggesting that each group influenced gut microbiota in a unique pattern.

At the phylum level, Firmicutes and Bacteroidetes were dominant in all the groups, accounting for a proportion of more than 75% of taxonomic units. Other phyla consisted of Proteobacteria, Spirochaetes, Candidatus Saccharibacteria, Deferribacteres, Tenericutes, Actinobacteria, and Verrucomicrobia. The administration of D-galactose for 45 days resulted in an increase in Firmicutes (D-galactose vs. control, 55.82% vs. 41.24%) and a decrease in Bacteroidetes (D-galactose vs. control, 31.86% vs. 33.72%) as compared to the control group mice (Figure 5B). Similarly, a previous study [55] reported an increase in Firmicutes and a decrease in Bacteroides after D-galactose injection for two months. The supplementation of EPSRam12 reversed the changes in Firmicutes and Bacteroidetes, decreased the increased abundance of Proteobacteria, and increased the decreased proportion of Spirochaetes caused by the injection of D-galactose (Figure 5B). The abundance of Proteobacteria has been reported as a potential diagnostic marker for dysbiosis and other metabolic diseases [56]. Spirochaetes have been reported to degrade polymers like xylan, pectin arabinogalactan, and hemicellulose and have also been known to synthesize short-chain fatty acids from plant polysaccharides [57,58]. Based on the above inference, Spirochaetes may utilize EPSRam12 and Beta-glucan, resulting in an increase in the proportion of Spirochaetes in the EPS and Beta-glucan group. The above results indicate that EPSRam12 improved the gut microbiota dysbiosis with the injection of D-galactose at the phylum level by decreasing the pathogens such as Proteobacteria and increasing the SCFA-producing bacteria such as Spirochaetes.

The analysis at the family level showed that Ruminococcaceae and Lachnospiraceae increased with the supplementation of EPSRam12 in a dose-dependent manner (Figure 5C); these have been reported as the most prevalent families in the gut of healthy human subjects, and are involved in the degradation of complex carbohydrates to produce SCFAs [57]. An increased Prevotellaceae-dominated microbiota in EPSRam12-supplemented groups was observed (Figure 5C–E), which has a positive correlation with dietary carbohydrate and is characterized by high propionate production, which can be explained by the SCFA-producing ability of *Prevotella* spp. [59,60]. Furthermore, an increase in the number of members of the Porphyromonadaceae family with the supplementation of EPSRam12 was observed, which has been reported to include SCFA-producing bacteria [57]. In contrast, supplementation with EPSRam12 decreased the abundance of the Helicobacteraceae family, which is dominated by the *Helicobacter* genus (Figure 5C–E), which has been reported to induce either direct cytotoxic and pro-inflammatory effects, or indirect effects by affecting the brain–gut axis [10]. 

At the genus level, the abundance of bacterial genera varied across all groups (Figure 5E). The relative abundances of *Prevotella*, *Clostridium_XlVa*, *Alistipes*, *Treponema*, *Saccharibacteria_incertae_sedis*, *Mucispirillum*, *Intestinimonas*, *Acetatifactor*, *Oscillibacter*, *Desulfovibrio*, and *Clostridium*_IV were reduced in the D-galactose group compared with that of the control group, but these genera were improved with the supplementation of EPSRam12. The abundance of genus *Prevotella* may be measured by its capacity to digest complex carbohydrates and fibers [59], resulting in higher production of total SCFAs (propionate and butyrate) [60], which may explain why *Prevotella* was selectively increased with EPSRam12 treatment in the current study. The anti-inflammatory effects and maintenance of intestinal health of EPSRam12 can be explained by the enrichment of *Clostridium* IV and XIVa, which has been reported to be influenced by dietary polysaccharides such as inulin, oligofructose, arabinoxylan, guar gum, and resistant starch to either directly cause the production of butyrate, which can exert anti-inflammation effects and maintain intestinal health, or indirectly facilitate acetate production from bifidobacterial strain fermentation, providing more substrates for *Clostridium* species to produce butyrate [61]. An increase in the abundance of *Alistipes* has been reported to alleviate colitis and possibly inflammatory bowel disease (IBD) in patients by improving the colon mucus barrier [62,63]. The abundance of *Treponema* has been positively correlated with a diet high in fiber content and with the ability to ferment polysaccharides including xylan and cellulose using carbohydrate-active enzymes to produce high levels of SCFAs, which play a protective role against gut inflammation [64]. *Intestinimonas*, a butyrate-producing genus found in the mouse intestine, has been shown to increase with polysaccharide diets such as *Lycium barbarum* polysaccharide, and oligosaccharides from wheat bran [65]. The *Acetatifactor* genus has been known to produce acetate and butyrate in the gut [66] and has also been reported to decrease with high-fat and high-calorie diets, while increasing with seaweed extracts (*Codium fragile*) [67]. *Oscillibacter* has been reported as a valerate producer, which has been positively correlated with the resistant starch and non-starch polysaccharides diets, that participate in T-cell differentiation by promoting and maintaining IL-10-producing Treg cells [67,68]. It has been demonstrated that higher abundance of *Clostridium* XIVa and *Oscillibacter* have anti-inflammatory properties and are crucial for preserving mucosal homeostasis [69]. The *Desulfovibrio* genus has been found to have a negative correlation with pro-inflammatory cytokines, and a positive correlation with serum acetic acid levels [70]. Increased *Desulfovibrio* abundance, particularly acetic acid production, has been reported with Astragalus polysaccharides obtained from herbal medicine, *Astragalus mongholicus* Bunge [70]. *Helicobacter* has been reported to induce several cytokines and chemokines, such as TNF-α, IL-6, IL-8, and histamine, which may disrupt the blood-brain barrier, leading to neurodegenerative diseases of the brain [10]. The inhibition of increased *Helicobacter* in the D-galactose group with the supplementation of EPSRam12 in a dose-dependent manner suggests the protective effect of EPSRam12 on the inflammation and neurodegeneration of the brains of mice. 

The principal coordinate plot of β-diversity based on weighted UniFrac distance, which considers the relative abundance of species/taxa shared between groups, represented 51.64% of total variance on the horizontal axis (PCoA1) and 32.64% on the vertical axis (PCoA2), representing the degree of difference between groups (Figure S1A). The distances between EPS25, EPS50, and Beta-glucan groups were shorter compared to the control group, whereas the D-galactose group showed the farthest distance compared to the control group, followed by the Stress-control group, along the PCoA1 axis, showing the recovery effects of EPSRam12 and positive control Beta-glucan. The principal coordinate plot based on unweighted UniFrac distance, which evaluates presence or absence, represented 38.26% of total variance on the horizontal axis (PCoA1) and 23.59% on the vertical axis (PCoA2) (Figure S1B). EPS25, Beta-glucan, and EPS50 groups had a longer distance from the control group along the PCoA1 axis, indicating different doses of EPSRam12 and Beta-glucan influenced gut microbiota in a unique pattern.

### 3.8. Short-Chain Fatty Acid Analysis in Mice Treated with EPSRam12

**Figure 6:**
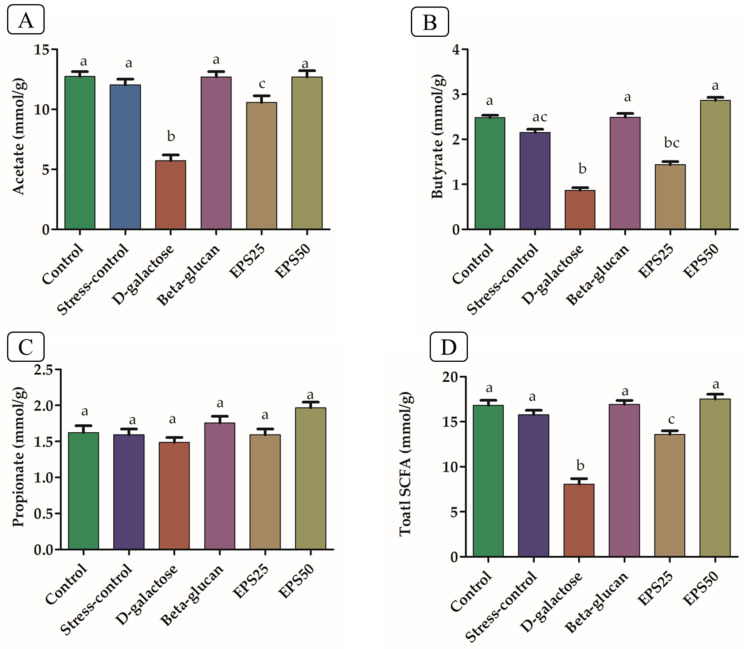
Short-chain fatty acid content in cecal samples of mice. (**A**) Acetate, (**B**) Butyrate, (**C**) Propionate, and (**D**) Total short-chain fatty acids (SCFA). Values are means ± SEMs. Data were presented as mean ± SEM. Data were analyzed using One-way ANOVA with Bonferroni post-test. Values with dissimilar superscript alphabets (a–c) indicate significant differences (*p* < 0.05) among the treatment groups.

The SCFA-producing genera *Prevotella*, *Clostridium* cluster IV and XIVa, *Treponema, Acetatifactor, Desulfovibrio, Mucispirillum, Intestinimonas,* and *Saccharibacteria_incertae_sedis*, which are correlated with diets high in fiber, increased with the supplementation of EPSRam12. Accordingly, we also measured the concentrations of acetic, propionic, and butyric acid in cecal samples of mice. The concentrations of acetate, butyrate, and total SCFAs increased significantly (*p* < 0.05) with the supplementation of EPSRam12 and Beta-glucan, compared with the respective decrease in SCFAs in the D-galactose group (Figure 6). The concentration of propionic acid between these groups did not differ significantly (*p* > 0.05). In line with this finding, the supplementation of fructo-oligosaccharide has been reported to increase cecal acetate and substantially increase *n*-butyrate and total SCFA concentrations in comparison to the corresponding fatty acid in the D-galactose group [71]. The supplementation of EPS produced by *Lactiplantibacillus plantarum* YW11 has been reported to increase the concentrations of acetic and butyric acids, compared with the D-galactose–treated group [52]. 

Polysaccharides have been proved to contribute significantly to the metabolic abilities of gut microbiota, such as the production of SCFAs. Polysaccharides and the production of SCFAs by the gut microbiota have been reported to improve mucus secretion and increase tight-junction protein expression [72,73]. Metabolites produced by gut microbiota have also been shown to improve cognitive function through modulation of the brain–gut axis [74]. A polysaccharide derived from *Polygonatum sibiricum* has been reported to reduce hippocampal oxidative stress, activation of NF-κB, inflammatory response in lipopolysaccharide, and chronic unpredictable mild stress-induced depression in models [75]. A polysaccharide derived from Schisandra Chinensis Fructus has been shown to ameliorate neuroinflammation in a mouse model of Alzheimer’s disease by lowering the expression of pro-inflammatory cytokines and regulating the NF-κB /MAPK pathway [76].

In addition, the ability of some polysaccharides to bind various micronutrients such as iron, calcium, and zinc has also been reported, and some of these micronutrients have been confirmed to prevent gut infection by inhibiting pathogens [72]. Furthermore, the production of SCFAs by gut microbiota may increase mineral solubility through acidification, increasing micronutrient bioavailability [72]. In this context, *L. rhamnosus* Ram12 has previously been reported to bind to ferrous sulphate at a rate of 61.63 0.67% [77]. This demonstrated EPSRam12’s superior ability to modulate the microbiota–gut–brain axis. Together, these findings might suggest that EPSRam12 supplementation may modulate the gut microbiota by increasing SCFA-producing bacteria and decreasing pathogens. The increased production of SCFAs in the intestine may be responsible for improving gut barrier integrity as well as decreasing oxidation and neuroinflammation in the brain. These findings and assumptions suggest that EPSRam12 may have a positive effect on the microbiota–gut–brain axis.

## 4. Conclusions

This study presents new evidence supporting the “microbiota-gut-brain axis” hypothesis that EPSRam12 plays a vital role in reducing brain injury, improving barrier integrity, and enriching production of SCFAs and modulating gut microbiota altered by D-galactose. EPSRam12 reduces brain injury by increasing the antioxidant status and anti-inflammatory effect of brain tissues via NF-κB signaling. EPSRam12 enhances intestinal epithelial barrier integrity by strengthening tight-junction mRNA expression and increasing the content of SCFAs. The SCFAs can directly or indirectly mediate microbiota–gut–brain interactions with gut–brain signaling pathways such as immune, endocrine, neural, and humoral pathways. Additionally, SCFAs may decrease neuroinflammation in the brain by influencing glial cell morphology and function, as well as by improving neuronal homeostasis and function. Furthermore, EPSRam12 treatment increases the abundance of SCFA-producing microbial genera such as *Prevotella*, *Clostridium*, *Intestinimonas*, and *Acetatifactor*, while decreasing the number of harmful bacteria such as *Helicobacter*. These findings demonstrate the significance of EPSRam12 in the alleviation of D-galactose-induced brain injury and altered gut microbiota. Furthermore, the findings highlight the potential utility of exopolysaccharide in a variety of pharmaceutical and other industrial applications. Further research should examine the functional mechanism(s) of EPSRam12 involved in the microbiota–gut–brain axis, and the metabolites produced by the gut microbiota with the supplementation of EPSRam12. The beneficial role of EPSRam12 in human trials should also be investigated further.

## Figures and Tables

**Table 1:** Real-time PCR primer sequences used in the study.

Gene	Primer Sequence	Product Size (bp)
ZO-1	F: 5′-CCAGCAACTTTCAGACCACC-3′ R: 5′-TTGTGTACGGCTTTGGTGTG-3′	154
Claudin	F: 5′-TGCACAGAGAGCAAGGGTATAG-3′ R: 5′-GAGCCGATCCATCCCAGAGA-3′	193
Occludin	F: 5′-GCTTACAGGCAGAACTAGACG-3′ R: 5′-TCTGCAGATCCCTTAACTTGC-3′	142
P65	F: 5′-TCTTCTTGCTGTGCGACAAG-3′ R: 5′-GCATGGAGACTCGAACAGGA-3′	177
INOS	F: 5′-ACAGGAACCTACCAGCTCAC-3′ R: 5′-CGACCTGATGTTGCCATTGT-3′	201
COX2	F: 5′-AGGTCATTGGTGGAGAGGTG-3′ R: 5′-CCTGCTTGAGTATGTCGCAC-3′	192
β-actin	F: 5′-AGAGGGAAATCGTGCGTGAC-3′ R: 5′-CAATAGTGATGACCTGGCCGT-3′	138
GAPDH	F: 5′-GCAAGAGAGAGGCCCTCAG-3′ R: 5′-TGTGAGGGAGATGCTCAGTG-3′	74

**Table 2:** Alpha-diversity analysis of the intestinal microbiota in mice.

Group	Number of ASVs	Chao1 Index	Shannon Index
Control	22	675	3.56
Stress-control	24	579	3.25
D-galactose	21	725	3.18
Beta-glucan	17	797	3.77
EPS25	15	640	3.39
EPS50	28	647	3.58

## Data Availability

Not applicable.

## Supplementary Materials

The following supporting information can be downloaded at: https://www.mdpi.com/article/10.3390/microorganisms10102046/s1, Figure S1: Effects of EPSRam12 on β-diversity in the gut microbiota composition of mice. (**A**) Principal coordinate plot based on weighted UniFrac distance. (**B**) Principal coordinate plot based on unweighted UniFrac distance.
