# Supplementary material for: Lacticaseibacillus rhamnosus-Derived Exopolysaccharide Attenuates D-Galactose-Induced Oxidative Stress and Inflammatory Brain Injury and Modulates Gut Microbiota in a Mouse Model
Source: Microorganisms. 2022 Oct 17;10(10):2046. doi: 10.3390/microorganisms10102046 (PMC9611687; doi:10.3390/microorganisms10102046)

Figure S1. Effects of EPSRam12 on β-diversity in the gut microbiota beta-diversity. (A) Principal coordinate plot based on weighted UniFrac distance. (B) Principal coordinate plot based on unweighted UniFrac distance.

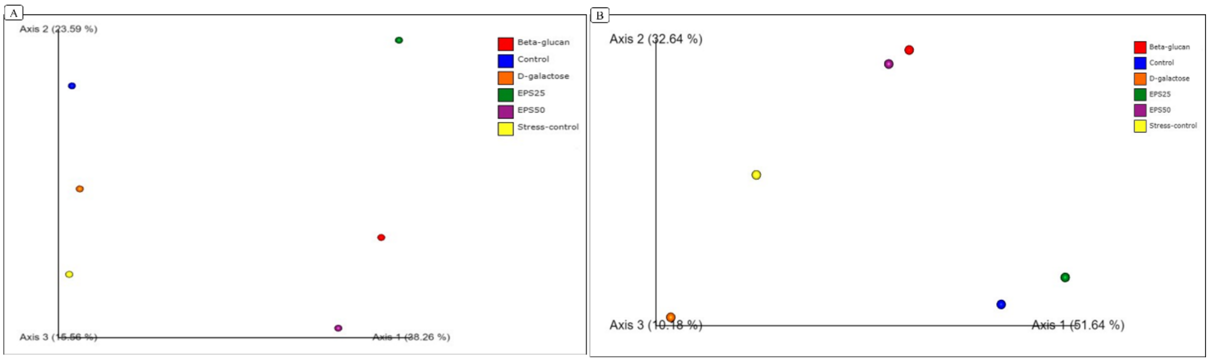

Supplement: Supplementary file 1 [file microorganisms-10-02046-s001.zip › microorganisms-1975507-supplementary.pdf]
